# QTL/Segment Mapping and Candidate Gene Analysis for Oil Content Using a Wild Soybean Chromosome Segment Substitution Line Population

**DOI:** 10.3390/plants15020177

**Published:** 2026-01-06

**Authors:** Cheng Liu, Jinxing Ren, Huiwen Wen, Changgeng Zhen, Wei Han, Xianlian Chen, Jianbo He, Fangdong Liu, Lei Sun, Guangnan Xing, Jinming Zhao, Junyi Gai, Wubin Wang

**Affiliations:** Jiangsu Key Laboratory of Soybean Biotechnology and Intelligent Breeding, Soybean Research Institute, National Innovation Platform for Soybean Breeding and Industry-Education Integration, Jiangsu Collaborative Innovation Center for Modern Crop Production, Sanya Institute of Nanjing Agricultural University, Nanjing Agricultural University, Nanjing 210095, China; liucheng@njau.edu.cn (C.L.); rjx282517@163.com (J.R.); 17835424958@163.com (H.W.); zhenchanggeng@stu.njau.edu.cn (C.Z.); hanwei2078125@163.com (W.H.); xlchen5200@163.com (X.C.); hjb@njau.edu.cn (J.H.); 2022002@njau.edu.cn (F.L.); sunlei@njau.edu.cn (L.S.); xinggn@njau.edu.cn (G.X.); jmz3000@126.com (J.Z.)

**Keywords:** wild soybean, chromosome segment substitution line, oil content, QTL, candidate gene

## Abstract

Annual wild soybean, the ancestor of cultivated soybean, underwent a significant increase in seed oil content during domestication. To elucidate the genetic basis of this change, a chromosome segment substitution line population (177 lines) constructed with cultivated soybean *NN1138-2* as recipient and wild soybean *N24852* as donor was used in this study. Phenotypic evaluation across three distinct environments led to the identification of two major QTL/segments, *qOC14* on chromosome 14 and *qOC20* on chromosome 20, which collectively explained 39.46% of the phenotypic variation, with individual contributions of 17.87% and 21.59%, respectively. Both wild alleles exhibited negative additive effects, with values of −0.35% and −0.42%, respectively, consistent with the inherently low oil content of wild soybeans. Leveraging transcriptome and genome data from the two parents, two candidate genes were predicted. Notably, *Glyma.14G179800* is a novel candidate gene encoding a PHD-type zinc finger domain-containing protein, and the hap-A haplotype exhibits a positive effect on oil content. In contrast, *Glyma.20G085100* is a reported *POWR1* gene, known to regulate protein and oil content. Our findings not only validate the role of known gene but, more importantly, unveil a new candidate gene, offering valuable genetic resources and theoretical targets for molecular breeding of high-oil soybean.

## 1. Introduction

Soybean (*Glycine max*) is a vital global crop, contributing approximately 27% of the world’s vegetable oil supply. It is extensively utilized in food, feed, and various industrial applications [1]. The primary component of soybean oil is triglycerides, characterized by a fatty acid profile that typically includes about 10% palmitic acid (C16:0), 4% stearic acid (C18:0), 18% oleic acid (C18:1), 55% linoleic acid (C18:2), and 13% linolenic acid (C18:3). This composition results in a high ratio of unsaturated fatty acids (up to 85%), which confers significant health benefits, such as reducing blood cholesterol levels and lowering the risk of coronary heart disease [2]. Consequently, enhancing both the oil content and fatty acid quality remains a central objective in soybean breeding programs.

Cultivated soybean was domesticated from its wild progenitor, annual wild soybean (*Glycine. soja*), through prolonged natural and artificial selection [3]. This domestication process led to fundamental improvements in key agronomic traits, with one of the most notable being a substantial increase in seed oil content. Wild soybeans are typically characterized by small seeds, low oil content (approximately 10%), high protein content, pod shattering, and a vining growth habit. In contrast, cultivated soybeans exhibit larger seeds, significantly higher oil content (about 18–22%), superior commercial quality, and an upright growth structure. Therefore, elucidating the genetic architecture underlying the transition from low-oil wild soybeans to high-oil cultivated varieties is of immense value for understanding soybean evolutionary history and advancing molecular design breeding for soybean improvement.

Soybean oil content is a complex quantitative trait controlled by polygenes and significantly influenced by environmental conditions. Quantitative trait locus (QTL) mapping has been a traditional and effective approach for dissecting the genetic architecture of such traits. To date, the SoyBase database (http://www.soybase.org, accessed on 2 October 2025) has cataloged more than 324 QTLs associated with oil content, distributed across all 20 soybean chromosomes [4,5]. These findings underscore that oil content is a typical polygenic trait whose expression is susceptible to environmental variation. However, conventional linkage mapping or genome-wide association studies (GWASs) based on natural populations are often confounded by population structure and genetic background noise, which can limit mapping accuracy, increase false-positive rates, and complicate subsequent gene cloning.

To overcome these limitations, chromosome segment substitution line (CSSL) populations have been developed. CSSL populations are constructed through multiple rounds of backcrossing coupled with molecular marker-assisted selection. Each line in such a population carries one or a few substituted segments from a donor parent against a uniform genetic background of the recurrent parent. This design effectively minimizes genetic background noise, allowing phenotypic differences to be unambiguously attributed to specific substituted segments. Thus, CSSL populations are ideal materials for high-precision QTL mapping and gene cloning. To exploit the rich genetic diversity present in wild soybeans, our research group developed a CSSL population (*SojaCSSLP5*) using wild soybean *N24852* (low oil content, 10.52%) as the donor and cultivated soybean *NN1138-2* (high oil content, 19.79%) as the recurrent parent. This population provides comprehensive coverage of the wild soybean genome [6,7,8,9,10], offering a powerful foundation for in-depth analysis of the molecular mechanisms driving soybean oil content evolution.

Although numerous QTLs for oil content have been mapped, only a limited number of causative genes have been successfully cloned and functionally validated in soybean. The reported genes are involved in diverse biological processes, including oil synthesis, transport, and transcriptional regulation. For instance, natural variation in the sugar transporter *SWEET39* leads to differences in oil content among soybeans [11]; *GmST05* regulates seed size and oil content by modulating the transcription of *GmSWEET10a* [12]; transcription factors *GmWRI1a* and *GmZF351* promote oil accumulation by activating the expression of lipid synthesis genes [13]; the transcription factor *GmVOZ1A* regulates soybean oil synthesis and interacts with *GmWRI1a* to upregulate the expression of *GmACBP6a*, promoting oil biosynthesis [14]; and *GmFATA1B* and *GmFA09* have been confirmed as key genes regulating oil content and fatty acid composition [1,15]. Additionally, *POWR1* (corresponding to *Glyma.20G085100* in this study) is a key domestication gene whose deletion in the CCT domain significantly affects seed protein content and oil content, as well as yield [16]. *STI*, which encodes a UDP-D-glucuronic acid 4-epimerase, has been shown to increase seed oil content [17]. These studies collectively reveal the intricate network governing oil metabolism in soybeans.

Based on above background, the present study leverages the high-precision *SojaCSSLP5* population to achieve two primary objectives: (1) to accurately identify wild soybean chromosome segments that significantly contribute to oil content through multi-environment phenotypic evaluation and elucidate their genetic effects; (2) to integrate genomic and transcriptomic data to systematically screen and functionally annotate genes within the mapped intervals, thereby identifying key candidate genes to provide direct targets for subsequent functional validation and molecular breeding of high-oil soybeans.

## 2. Results

### 2.1. Phenotypic Variation of Oil Content in the SojaCSSLP5

**Figure 1:**
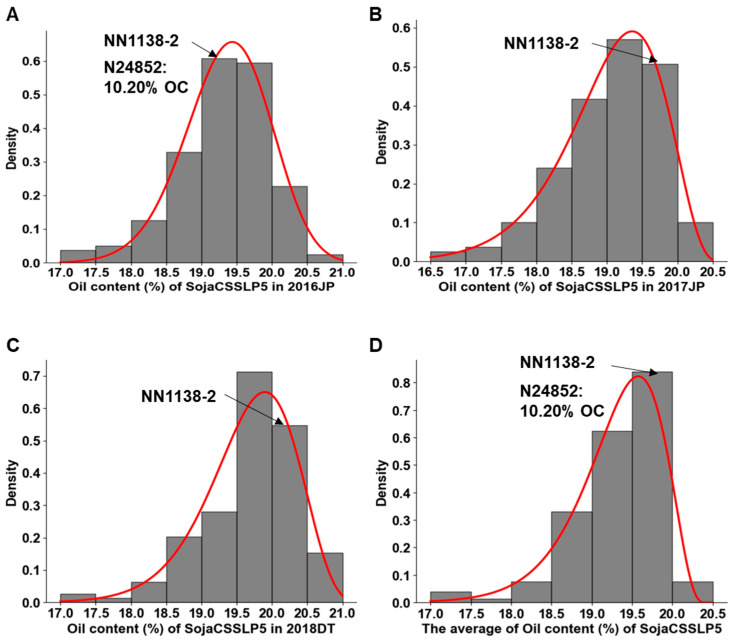
Frequency distribution of oil content (%) in the *SojaCSSLP5* Across Three Environments. (**A**–**D**) oil content of *SojaCSSLP5* in 2016JP (Jiangpu Experimental Station, summer 2016), 2017JP (Jiangpu Experimental Station, summer 2017), 2018DT (Dangtu Experimental Station, summer 2018) and average across three environments, respectively. OC, oil content. The red curve represents the beta fitting curve.

A comprehensive evaluation of oil content was conducted for the *SojaCSSLP5* and its parental lines across three distinct environments (2016JP, 2017JP, and 2018DT). The results revealed substantial phenotypic variation, as summarized in Table 1 and illustrated in Figure 1. In 2016JP, a significant difference in oil content was observed between the cultivated soybean parent, *NN1138-2* (19.29%), and the wild soybean parent, *N24852* (10.20%). The *SojaCSSLP5* population exhibited a broad range of oil content across all three environments: 17.16–20.58% in 2016JP, 16.72–20.31% in 2017JP, and 17.30–20.90% in 2018DT. This wide variation indicates that the introgression of wild soybean chromosome segments induced significant diversity in oil content within the population. The mean oil content of the population was 19.31%, 19.06%, and 19.68% in the three environments, respectively, closely aligning with that of the recurrent parent, *NN1138-2*. The absolute value of skewness (−0.73–−1.14) and kurtosis (0.97–1.96) indicate that the data conforms to a left skewed distribution in *SojaCSSLP5* population. The frequency distribution showed that most lines regressed to the oil content level of the recurrent parent, which is a characteristic feature of a CSSL population. The coefficients of variation (CV) for oil content in all three environments were below 15%, indicating well-controlled experimental error and high data reliability. The broad-sense heritability (*h*^2^) estimates were 61.59%, 77.37%, and 70.54% for the individual environments, with an average of 68.42% across environments, suggesting that the variation in oil content is mainly genetically controlled.

A joint analysis of variance (ANOVA) for oil content across the three environments was performed, and the results are presented in Table 2. The analysis revealed highly significant effects (*p* < 0.0001) for environment, replicates within environments, lines (genotypes), and the genotype-by-environment interaction. These results confirm that although oil content is predominantly genetically determined, it is also significantly influenced by environmental conditions and their interaction with the genotype. Consequently, using the mean phenotypic value across multiple environments for subsequent QTL mapping effectively minimizes environmental interference and enhances mapping accuracy.

### 2.2. Identification of Wild Segments Related to Oil Content

**Figure 2:**
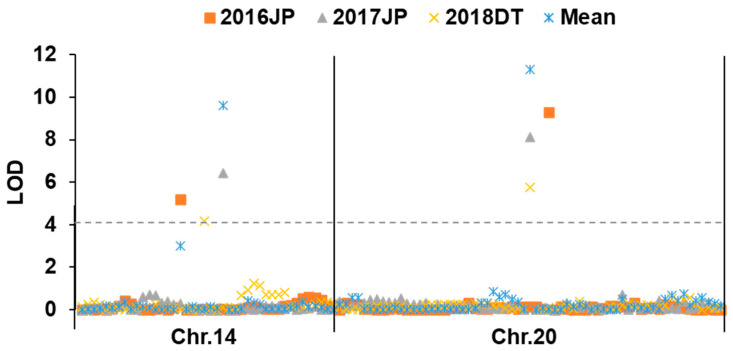
Identified QTL for oil content in *SojaCSSLP5*. The gray dashed line represents the threshold.

Using the multi-environment mean values of oil content, two significant wild segments/QTL associated with oil content were identified via QTL IciMapping software (version 4.0). The details of these QTL are provided in Table 3 and Figure 2. The first QTL, designated *qOC14*, was mapped to a 4.65 Mb interval (43.14–47.79 Mb) on chromosome 14, flanked by the marker Gm14_26. This locus achieved a highly significant LOD score of 9.60 and explained 17.87% of the phenotypic variation (PVE). The additive effect of the wild allele was −0.35%, indicating that the allele from wild soybean *N24852* reduces oil content. The second QTL, *qOC20*, was located on chromosome 20 within a 9.15 Mb interval (24.34–33.48 Mb), marked by Gm20_29. It exhibited a higher LOD score of 11.30 and a greater PVE of 21.59%. The wild allele at this locus also had a negative additive effect of −0.42%. Collectively, these two major QTLs accounted for 39.46% of the total phenotypic variation in oil content. Notably, the physical intervals of both *qOC14* and *qOC20* overlap with multiple previously reported oil content QTL in the SoyBase. For instance, *qOC14* overlaps with nine known QTL (e.g., *Seed oil 43-6*), while *qOC20* overlaps with eleven (e.g., *Seed oil 2-1*). This co-localization with independently mapped QTL cross-validates the reliability of our findings.

**Figure 3:**
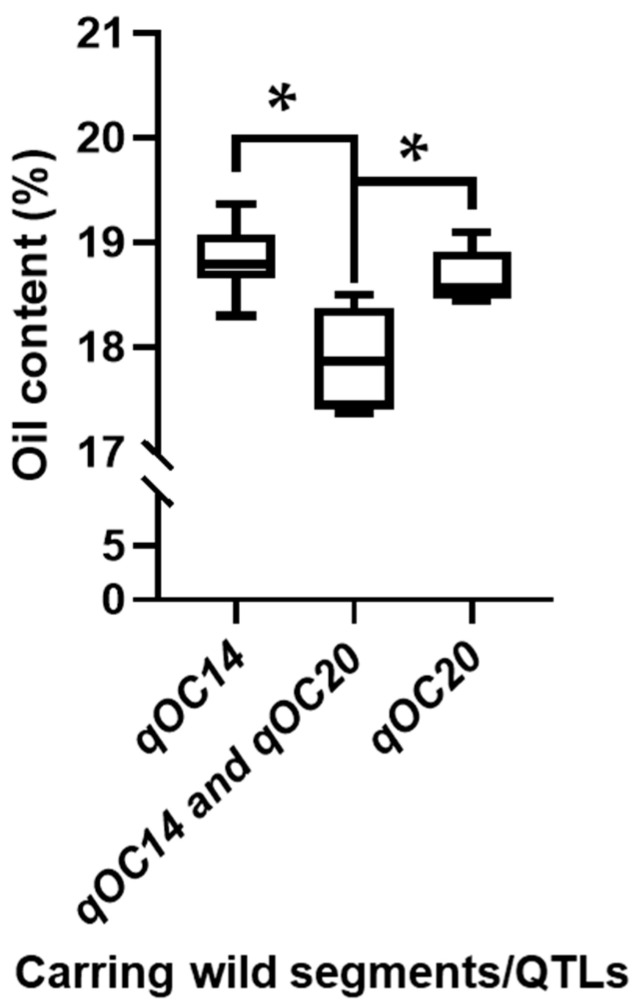
Differences in oil content (%) among CSSLs carrying wild segments of *qOC14* (Gm14_26) and *qOC20* (Gm20_29). *qOC14*, CSSLs with only single segment/QTL of *qOC14*. *qOC14* and *qOC20,* CSSLs with both segments/QTLs of *qOC14* and *qOC20*. *qOC20,* CSSLs with only single segment/QTL of *qOC20*. Significance: * *p* < 0.05 (*t*-test), showing additive effects of wild alleles.

To visually demonstrate the phenotypic effects of these QTL, a subgroup analysis was conducted on the CSSLs carrying the detected wild segment (Table 4). Within the population, 12 and 10 CSSLs carried the wild segments *Gm14_26* (*qOC14*) and *Gm20_29* (*qOC20*), respectively, in a total of 18 ones (some CSSLs carried both segments). With the exception of CSSL *L113*, the oil content of these lines was significantly lower than that of the recurrent parent, *NN1138-2*. Among them, four lines (*L092*, *L093*, *L163*, and *L174*) carried both wild segments/QTLs (*qOC14* and *qOC20*) simultaneously. These lines exhibited significantly lower oil content compared to lines carrying only a single wild segment (Table 4, Figure 3). This result, illustrated in Figure 3, suggests that *qOC14* and *qOC20* might have independent and additive genetic effects on reducing oil content.

### 2.3. Validation of Candidate Gene Related to Oil Content

**Figure 4:**
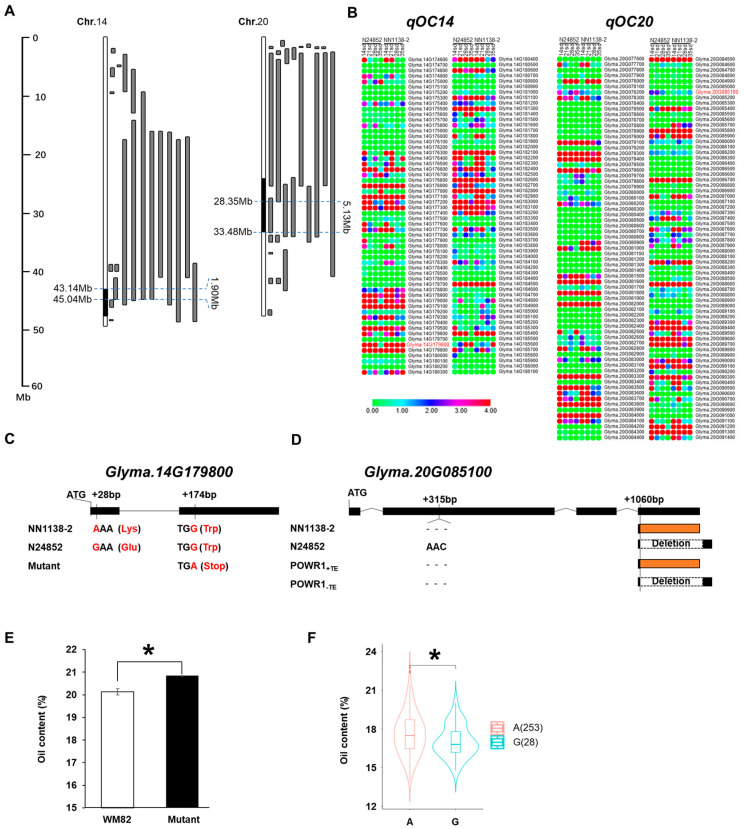
Identification of Candidate Genes for Oil Content in Soybean. (**A**) QTL interval refinement using SoyBase data; (**B**) Gene expression filtering (green circles: FPKM = 0); (**C**,**D**) Sequence variations in candidate genes. The dashed box represents the missing 321 bp sequence; (**E**,**F**) Validation via mutant and haplotype analysis. * *p* < 0.05.

A number of QTL related to soybean oil content have been reported previously. Specifically, SoyBase records 20 and 27 QTL on chromosomes 14 and 20, respectively. By integrating the physical intervals of the QTL mapped in this study with the positions of these previously reported QTL, the target regions were refined to 1.90 Mb (43.14–45.04 Mb) for *qOC14* and 5.13 Mb (28.35–33.48 Mb) for *qOC20*, which contained 116 and 169 annotated genes, respectively (Figure 4A).

A systematic candidate gene analysis was then performed. First, genes with no expression (FPKM = 0) in the seed RNA-seq data from both parents were filtered out (indicated by green circles in Figure 4B). Subsequently, based on the whole-genome resequencing data of *NN1138-2* and *N24852*, genes containing non-synonymous SNPs or InDels in their coding sequences (CDS) were prioritized. This integrated approach led to the identification of two high-confidence candidate genes: *Glyma.14G179800* within the *qOC14* interval and *Glyma.20G085100* within the *qOC20* interval.

*Glyma.14G179800* encodes a PHD-type zinc finger plant domain-containing protein. Sequencing revealed a non-synonymous SNP at the +28 bp position, a *G* base in the wild soybean (*N24852*) is mutated to an *A* base in the cultivated soybean (*NN1138-2*), resulting in an amino acid change from Glutamate (Glu) to Lysine (Lys) in the translated protein (Figure 4C, Table 5). Therefore, *Glyma.14G179800* was considered a novel candidate gene for the *qOC14* locus. *Glyma.20G085100*, encodes a protein containing a CCT motif. Sequence analysis identified a 3 bp InDel variation. More importantly, a sequence variation at the +1060 bp position in the wild soybean allele leads to 321 bp deletion and premature termination of the translated protein (Figure 4D, Table 5). This gene of the *NN1138-2* allele corresponds to the previously reported *POWR1_+TE_* gene, known to be associated with low protein content and high oil content.

Further preliminary validation strengthened the evidence for *Glyma.14G179800*. Analysis of oil content data from a soybean Williams 82 (WM82) TILLING mutant library showed that the mutant for *Glyma.14G179800* had an oil content of 20.84%, which was significantly higher than the 20.27% observed in the wild-type WM82, representing a 2.81% increase (Figure 4E). Furthermore, analysis of resequencing data from 381 cultivated soybean accessions revealed one SNP at the +28 bp position of *Glyma.14G179800*, defining two haplotypes: hap-A (genotype A, identical to *NN1138-2*) carried by 253 accessions, and hap-G (genotype G, identical to *N24852*) carried by 28 accessions. The oil content of accessions carrying the hap-A haplotype was significantly higher than that of those carrying the hap-G haplotype (Figure 4F). These results provide compelling evidence that *Glyma.14G179800* is a key candidate gene for oil content, with the hap-A allele representing a superior haplotype selected during soybean domestication and breeding.

## 3. Discussion

### 3.1. The Utility of CSSL Populations in Deciphering Domestication Traits

The present study underscores the significant utility of the *SojaCSSLP5* population in dissecting the genetic architecture underlying oil content, a key trait modified during soybean domestication. Compared to traditional linkage analysis or association mapping populations, CSSL populations offer distinct advantages for analyzing complex quantitative traits. Firstly, each line carries only one or few introgressed donor segments against a largely uniform recurrent parent background, which dramatically minimizes genetic background noise. This design enables more precise QTL mapping and facilitates the accurate analysis of epistatic interactions between QTL [30,31]. Secondly, such populations significantly streamline subsequent fine-mapping and map-based cloning efforts. Thirdly, when the donor parent is the wild progenitor, as in this case, the detected QTLs directly reflect allelic divergence that arose during domestication, making CSSLs an ideal genetic resource for studying domestication mechanisms. This approach has been successfully employed in other crops like rice and tomato [32,33].

In our study, using the wild soybean CSSL population, we identified two major QTL, *qOC14* and *qOC20*, associated with oil content. While we identified significant genetic loci associated with seed oil content, we recognize that this complex quantitative trait is substantially influenced by environmental factors (e.g., temperature, rainfall, soil nutrients). Our phenotypic data were collected from multiple but finite environments and seasons. Therefore, the observed phenotypes and the estimated effects of the identified quantitative trait loci (QTLs)/alleles might vary under different environmental conditions. This inherent genotype-by-environment interaction could affect the generalizability and consistent performance of the marker alleles identified here. The wild alleles at both loci exerted negative additive effects, consistent with the characteristically low oil content of wild soybeans. This finding confirms that these “low-oil” alleles were likely gradually eliminated or improved upon during domestication. Furthermore, CSSLs simultaneously carrying both wild segments exhibited significantly lower oil content than lines carrying only a single segment, indicating that the effects of these loci are additive and independent. This result provides a theoretical basis for further enhancing oil content through pyramiding favorable alleles in breeding programs.

### 3.2. The Complex Genetic Network Governing Soybean Oil Domestication

Based on the wild soybean CSSL population, this study successfully identified two segments/QTL contributing to oil content. A comparative analysis with previously mapped QTLs revealed substantial overlap, enhancing the credibility of our findings. The *qOC14* interval overlaps with nine reported QTL, including *Seed oil 43-6* [18], while the *qOC20* interval co-localizes with eleven known QTL, such as *Seed oil 2-1* [24]. The convergence of independent mapping studies on these genomic regions strongly cross-validates their importance in controlling oil content. However, collectively, these two major QTL explain only 39.46% of the total phenotypic variation. Given that the broad-sense heritability of oil content was estimated at 68.42%, approximately 28.96% of the genetic variation remains unexplained. This result clearly indicates that the domestication of soybean oil content involves a more complex genetic network than captured by these two loci. Beyond the genes identified here, it is likely that numerous minor-effect QTLs participate in various biological processes, including oil synthesis, degradation, transport, and carbon source supply, as well as potential epistatic interactions not captured by our additive model. Furthermore, the trait’s phenotypic complexity may encompass unmeasured biochemical sub-components with partially independent genetic regulation. In the future, we will construct a population of wild soybean chromosome single segment substitution lines with high-density mapping, which will not rely on mapping software and can discover more minor-effect QTLs. Additionally, a well-documented strong genetic negative correlation exists between oil content and other seed traits, particularly protein content and seed size. This trade-off relationship could be governed by pleiotropic genes like *POWR1* or result from intricate interactions between distinct regulatory modules. Future research employing larger populations, higher-resolution mapping strategies, and systems biology approaches will be essential to fully elucidate the comprehensive genetic network regulating soybean oil content.

### 3.3. Candidate Gene Prediction and Putative Molecular Mechanisms

Through an integrated analysis of multi-omics data, this study identified two high-confidence candidate genes within the mapped intervals. *Glyma.20G085100* (*POWR1*), with previously validated function, encodes a nucleic acid-binding protein containing a CCT domain. It is hypothesized to play a central role in regulating the balance between protein and oil metabolism by modulating the allocation of seed nutrients. Its mechanism may involve functioning as a transcriptional regulator that influences the expression of downstream genes related to either oil synthesis or protein accumulation [16]. Our results align with the findings of Goettel, Zhang, Li, Qiao, Jiang, Hou, Song, Pantalone, Song, Yu and An [16]: in the cultivated parent *NN1138-2*, a transposable element (TE) insertion truncates the CCT domain of *POWR1_+TE_*, which is associated with increased seed oil content and hundred-seed weight, alongside reduced protein content. However, in wild soybean *N24852*, a distinct allelic variation was identified, characterized by an insertion of “AAC” at the +315 bp position and the absence of the 321 bp TE, which differs from the *POWR1_-TE_*. The recurrence of this gene in our population not only validates its functional significance but also demonstrates the effectiveness of the *SojaCSSLP5* population for mining domestication-related genes.

This study proposes *Glyma.14G179800* as a novel candidate gene for oil content. It encodes a protein containing a PHD-type zinc finger domain. The PHD zinc finger domain is a recognized epigenetic “reader” module that can recognize specific histone modifications (e.g., H3K4me3) and subsequently recruit chromatin remodeling complexes to modulate gene transcription [34]. In plants, PHD proteins are known participants in various developmental processes and stress responses. For instance, the Arabidopsis *MS1* gene, which contains a PHD domain, regulates pollen development [35]; the rice *SAB23* gene affects submergence tolerance [36]; and soybean *GmPHD2* is associated with salt tolerance [37]. However, direct evidence linking PHD proteins to the regulation of lipid metabolism remains scarce. Although *Glyma.14G179800* emerged as a promising candidate within a stable QTL region, supported by haplotype and expression analysis, our study did not include direct functional validation to conclusively prove its role in regulating soybean oil content. Its annotation and co-expression pattern are strongly suggestive, but without experimental confirmation, a definitive causal relationship cannot be established. In future research, we will further identify this gene by constructing secondary populations for fine mapping. Then, the function of *Glyma.14G179800* was validated through gene editing, overexpression techniques, and ChIP-seq/qPCR. Although a direct link between a PHD protein and lipid metabolism is yet to be reported, we hypothesize that the identified PHD-type zinc finger protein (*Glyma.14G179800*) may function as an H3K4me3 ‘reader’ that integrates developmental or environmental signals to fine-tune oil biosynthesis. It could do so by: (i) Directly binding to the H3K4me3-enriched chromatin of key regulators like *GmWRI1a*, where it recruits histone acetyltransferases (e.g., HAC1) to further open chromatin, or interacts with the transcription initiation complex to enhance their transcription. (ii) Acting upstream in the hierarchy by regulating the chromatin state of master regulators (e.g., *LEC2*) that control the WRI1-ACBP network [14]. This model aligns with the known role of PHD proteins in maintaining cell identity and metabolic states during seed development and provides a clear epigenetic layer to the regulation of oil content. The key amino acid substitution (Glutamate to Lysine) between the wild and cultivated alleles might alter the protein’s conformation or its binding affinity for histone marks, DNA, or interaction partners, ultimately affecting the transcriptional activity of downstream target genes and leading to oil content variation. This hypothesis warrants further validation through subsequent experiments, including gene editing, overexpression, yeast two-hybrid assays, and chromatin immunoprecipitation (ChIP). By further determining the function of the gene through these techniques, it will have certain application value in modern soybean breeding.

## 4. Materials and Methods

### 4.1. Plant Materials

The plant materials utilized in this study consisted of the cultivated soybean (*Glycine max*) accession *NN1138-2*, the wild soybean (*Glycine soja*) accession *N24852*, and a derived wild soybean CSSL population designated *SojaCSSLP5*, which comprises 177 lines. The recurrent parent, *NN1138-2* (maturity group V), is an elite breeding line in southern China with an oil content of approximately 19.79%. This variety was subjected to whole-genome resequencing at an average depth of 11.39×. The donor parent, *N24852* (maturity group III), exhibits a significantly lower oil content of about 10.20%. Whole-genome resequencing of *N24852* was performed at an average depth of 9.51×. To precisely delineate the wild soybean substituted segments in each line, the entire *SojaCSSLP5* population underwent whole-genome resequencing at an average depth of 3.04×. This sequencing effort yielded 2,567,426 high-quality single nucleotide polymorphisms (SNPs) and enabled the definition of 1366 SNP linkage disequilibrium markers (SNPLDBs). The substituted segments collectively cover 99.74% of the wild soybean genome, constituting a near-complete genomic representation of the donor parent. Detailed methodologies for population construction and sequencing have been described previously [10].

### 4.2. Field Trial Design and Phenotypic Measurement

To ensure accurate evaluation of oil content, field trials were conducted across three distinct environments: summer 2016 at the Jiangpu Experimental Station of Nanjing Agricultural University (2016JP), summer 2017 at the same station (2017JP), and summer 2018 at the Dangtu Experimental Station of Nanjing Agricultural University (2018DT). A randomized complete block design was employed with three replications. Each plot consisted of a single row that was 1 m long, containing 10 plants, with a row spacing of 0.5 m. Standard agricultural practices were followed for field management. Seeds were harvested at full maturity on a per-plot basis. To standardize moisture content, the harvested seeds were dried in a constant-temperature oven at 32 °C for five consecutive days. Subsequently, high-quality seeds free from mechanical damage, broken seed coats, or mildew were selected. The oil content of these at least 200 seeds was directly measured using a Foss Infratec 1241 (Foss Tecator, Höganas, Sweden) that is a benchtop near-infrared (NIR) analyzer designed for the rapid, non-destructive analysis of whole grain samples and operates on the principle of near-infrared transmission spectroscopy. Phenotypic data for the wild soybean parental line *N24852* is presented only for the 2016JP environment. Data for the 2017JP and 2018DT are unavailable due to harvest failure caused by the line’s characteristic pod shattering and extremely small seed size under the prevailing natural conditions in those years, which prevented the collection of sufficient seed material for reliable oil content analysis using the FOSS NIR system.

### 4.3. Statistical Analysis

Descriptive statistics, including range, mean, skewness, kurtosis, and coefficient of variation (CV), were calculated for the oil content data from each environment and for the combined data across environments. Analysis of variance (ANOVA) was performed to assess the significance of various factors. The broad-sense heritability (*h*^2^) was estimated using the formula:

$$h^{2}=\frac{{\sigma^{2}}_{g}}{{\sigma^{2}}_{g}+\frac{{\sigma^{2}}_{ge}}{n}+\frac{{\sigma^{2}}_{e}}{nr}}$$

where *σ*^2^*_g_* represents the genotypic variance, *σ^2^_ge_* the genotype–environment interaction variance, *σ*^2^*_e_* the error variance, *n* the number of environments, and *r* the number of replicates. A joint ANOVA across multiple environments was conducted using the PROC MIXED procedure in SAS 9.4 software (SAS Institute, Cary, NC, USA) to evaluate the effects of environment, genotype, and their interaction on oil content. Student’s *t*-test was used to detect significant differences in data, including groups of CSSLs, mutant, and haplotype analysis.

### 4.4. Segment/QTL Mapping

The RSTEP-LRT-ADD model in QTL IciMapping 4.0 software was used to detect oil content-related chromosome segments/QTLs. This model is based on stepwise regression analysis and likelihood ratio test (LRT) for QTL detection, suitable for non-ideal CSSL populations. The significance threshold for LOD values was determined through 1000 permutation tests (*p* < 0.05). Detected QTLs were named according to international conventions, i.e., “q” + trait abbreviation (OC) + chromosome number. The additive effect value of a QTL indicates the direction and magnitude of the effect of carrying the wild segment allele on the phenotypic value.

The identification of segments/QTL associated with oil content was performed using the RSTEP-LRT-ADD model in QTL IciMapping software (version 4.0). This model is particularly suitable for non-ideal CSSL populations as it employs stepwise regression analysis combined with a likelihood ratio test (LRT) for QTL detection. The statistical significance threshold for logarithm of odds (LOD) scores was determined empirically through 1000 permutation tests, corresponding to a significance level of *p* < 0.05. Detected QTLs were named according to standard nomenclature: “q” followed by the trait abbreviation (OC) and the chromosome number. The additive effect value for each QTL indicates the direction and magnitude of the phenotypic effect conferred by the allele from wild soybean.

### 4.5. Candidate Gene Analysis

To analyze candidate genes related to oil content within the mapped regions, the following steps were performed: Firstly, the detected QTLs were integrated and co-localized with previously reported oil content QTLs in the SoyBase database to further narrow the target genomic intervals. Secondly, RNA-seq data from both parental lines (*NN1138-2* and *N24852*) across four critical seed development stages (14, 21, 28, and 35 days after flowering) from a previous study [38] were utilized to screen for genes exhibiting differential expression. Thirdly, based on the whole-genome resequencing data of the two parents [10], genes harboring non-synonymous SNPs or insertions/deletions were prioritized, as these variations are likely to alter protein amino acid sequences and potentially affect gene function. Finally, Gene Ontology and functional annotation analyses were conducted on the shortlisted candidate genes to predict their biological roles and identify the most promising key candidate genes associated with oil content within the mapped intervals.

## 5. Conclusions

This study successfully delineates the genetic basis of soybean oil content domestication by leveraging a genome-wide wild soybean chromosome segment substitution line population (*SojaCSSLP5*), integrated with multi-environment phenotypic data and genomic analyses. We identified two major QTLs, *qOC14* and *qOC20*, whose wild alleles exhibited negative additive effects, collectively explaining 39.46% of the phenotypic variation and confirming their pivotal roles in the domestication process. Our findings robustly validate the function of the known gene *POWR1* (*Glyma.20G085100*) within the *qOC20* interval in this population and, more significantly, unveil a novel candidate gene, *Glyma.14G179800*, within the *qOC14* locus. This gene, encoding a PHD-type zinc finger protein, suggests a potential epigenetic mechanism for oil accumulation. Genetic analysis confirmed the additive effects of these loci, as lines carrying both wild segments exhibited the lowest oil content, providing a clear strategy for marker-assisted pyramiding of favorable alleles. This work delivers valuable genetic resources and novel targets for the molecular breeding of high-oil soybean varieties.

## Figures and Tables

**Table 1:** Variation of oil content in the *SojaCSSLP5* along with its two parents.

Env.	Parents			*SojaCSSLP5*				
	*NN1138-2*	*N24852*	Range (%)	Mean (%)	Skewness	Kurtosis	CV (%)	*h*^2^ (%)
2016JP	19.29	10.20	17.16–20.58	19.31	−0.73	0.97	3.40	61.59
2017JP	19.65	-	16.72–20.31	19.06	−0.80	0.65	2.90	77.37
2018DT	20.27	-	17.30–20.90	19.68	−0.80	0.89	2.94	70.54
Mean	19.79	10.20	17.37–20.24	19.35	−1.14	1.96	3.08	68.42

Note: Environments: 2016JP (Jiangpu Experimental Station, summer 2016), 2017JP (Jiangpu Experimental Station, summer 2017), 2018DT (Dangtu Experimental Station, summer 2018). Mean: average across three environments. CV: coefficient of variation (%). *h*^2^: broad-sense heritability (%). Data presented as percentage oil content. “-” indicates data not available.

**Table 2:** Joint analysis of variance (ANOVA) for oil content in the *SojaCSSLP5*.

Source of Variation	DF	MS	F Value	*p*
Line	154	2.08	3.07	<0.0001
Error (Line)	311.78	0.67		
Env.	2	43.63	6.19	0.0307
Error (Env.)	6.57	7.04		
Rep (Env.)	6	6.76	18.94	<0.0001
Line × Env.	309	0.68	1.91	<0.0001
Error (MS)	863	0.36		

DF: degrees of freedom; MS: mean square; F value: F-statistic from ANOVA; *p*: significance level (*p* < 0.0001 indicates highly significant effects). Model specifications: Genotype (Line) treated as fixed effect; Environment (Env.), and Rep (Env.) treated as random effect. Replicate (Rep) effects are nested within environments.

**Table 3:** Major QTL/Segments Associated with Oil Content Identified in the *SojaCSSLP5*.

QTL	Marker	Genome Region	Size of Region	LOD	PVE (%)	Add	Reported QTL
*qOC14*	Gm14_26	43,138,590–47,789,260	4.65	9.60	17.87	−0.35	*Seed oil 43-6* [18], *Seed oil 28-1* [19], *Seed oil 43-5* [18], *Seed oil 34-2* [20], *Seed oil 24-17* [21], *Seed oil 37-4* [22], *Seed oil 43-2* [18], *Seed oil 30-4* [23], *Seed oil 43-3* [18]
*qOC20*	Gm20_29	24,336,033–33,481,640	9.15	11.30	21.59	−0.42	*Seed oil 2-1* [24], *Seed oil 2-2* [24], *Seed oil 11-1* [25], *Seed oil 12-1* [25], *Seed oil 13-4* [26], *Seed oil 15-1* [27], *Seed oil 24-29* [21], *Seed oil 24-30* [21], *Seed oil 32-3* [28], *Seed oil 42-19* [29], *Seed oil 43-17* [18]

QTLs named as ‘q’ + trait abbreviation (OC) + chromosome number. Marker: flanking marker for QTL interval. Genome region: physical position in base pairs (bp). LOD: logarithm of odds score (threshold set via 1000 permutations, *p* < 0.05). PVE: percent phenotypic variation explained by the QTL. Add: additive effect of wild allele (negative values indicate reduction in oil content). Reported QTLs are from SoyBase database.

**Table 4:** Comparison of oil content among CSSL carrying wild segments Gm14_26 and Gm20_29.

Line	Oil Content	Gm14_26	Gm20_29	Significance
*NN1138-2*	19.79	A	A	
*L005*	18.87	B	A	*
*L047*	18.66	B	A	**
*L050*	18.73	B	A	*
*L086*	18.79	B	A	*
*L113*	19.37	B	A	ns
*L144*	18.30	B	A	**
*L150*	19.08	B	A	*
*L182*	17.95	B	A	***
*L092*	18.50	B	B	**
*L093*	18.26	B	B	**
*L163*	17.37	B	B	***
*L174*	17.87	B	B	***
*L049*	18.45	A	B	**
*L076*	18.85	A	B	*
*L151*	18.51	A	B	**
*L157*	18.48	A	B	**
*L173*	18.64	A	B	*
*L176*	19.10	A	B	**

Genotype notation: A: homozygous for recurrent parent *NN1138-2* allele; B: homozygous for wild soybean *N24852* allele. Significance levels: ns, not significant; * *p* < 0.05; ** *p* < 0.01; ***, *p* < 0.001 (based on *t*-test comparison with recurrent parent). Data show oil content (%) averaged across three environments.

**Table 5:** Candidate genes for oil content predicted from expression differences between *NN1138-2* and *N24852*.

Gene	Function	Parents	Expression Difference (FPKM)				
			14seed	21seed	28seed	35seed	Leaf
*Glyma.14G179800*	PHD-type zinc finger plants domain-containing protein	*NN1138-2*	16.35	7.63	6.09	6.84	16.35
		*N24852*	19.02	12.89	5.42	0.55	19.02
*Glyma.20G085100*	CCT motif-containing protein	*NN1138-2*	0.59	1.13	0.57	1.31	0.12
		*N24852*	2.23	2.26	1.45	0.19	0.64

Gene function based on domain annotation: PHD-type zinc finger domain involved in epigenetic regulation; CCT motif associated with nutrient balance. Expression values: FPKM (Fragments Per Kilobase per Million) from RNA-seq data at seed development stages (14, 21, 28, 35 days after flowering) and leaf tissue. Non-synonymous SNPs/InDels were prioritized from resequencing data.

## Data Availability

Data are contained within the article.

## Abbreviations

The following abbreviations are used in this manuscript:

CSSL	Chromosome Segment Substitution Line
LOD	Logarithm of odds
PVE	Percentage of phenotypic variation explained by individual QTL
QTL	Quantitative trait locus
SNP	Single nucleotide polymorphism
